# A Dual-Layer Hybrid-A* Path Planning Algorithm for Unstructured Environments Based on Phase Windows

**DOI:** 10.3390/s26010043

**Published:** 2025-12-20

**Authors:** Tianxiao Zhu, Ziyu Xu, Rujiang Zhu, Wei Zhang, Zhonghua Miao

**Affiliations:** 1 School of Mechatronic Engineering and Automation, Shanghai University, Shanghai 200444, China; 2 State Key Laboratory of Agricultural Equipment Technology, Beijing 100083, China

**Keywords:** unstructured environments, Hybrid-A* algorithm, mobile robot, path planning, quadratic programming

## Abstract

In mobile robotics, path planning enables autonomous navigation to specified destinations. However, complex terrain can lead to excessive tilting or even overturning, compromising stability and safety. Traditional path-planning algorithms often fail to fully account for dynamic terrain variations and robot motion constraints. To address these limitations, this paper proposes the novel dual-layer Hybrid-A* algorithm, enhanced with dynamic phase windows. This approach represents a significant innovation by integrating real-time feedback mechanisms and adaptive adjustments to phase windows, enabling continuous path refinement in response to both environmental changes and robot motion limitations. The guidance layer introduces a bicubic interpolation-based super-resolution technique to refine elevation maps, offering more accurate posture estimation. In the planning layer, we propose the dynamic use of multiple cost functions, an adaptive expansion radius, pruning strategies, and a phase-window activation mechanism, effectively addressing the computational challenges posed by large search spaces. The integration of these strategies allows the algorithm to outperform traditional methods, particularly in unstructured environments with complex terrain. Experimental results demonstrate the effectiveness of the proposed method in generating optimized paths that satisfy robot motion constraints, ensuring both efficiency and safety in real-world applications.

## 1. Introduction

Mobile robots have been extensively deployed in domains such as logistics, agriculture, defense, and aerospace, where they increasingly replace humans in repetitive, hazardous, or heavy-load tasks [1,2]. Global path planning enables mobile robots to autonomously construct feasible operational routes based on global environmental maps [3,4]. Consequently, path-planning approaches must address two fundamental challenges: generating accurate environmental representations and performing independent path searches within these maps [5].

These challenges directly influence the operational performance and efficiency of mobile robots. In general, a feasible planned path is expected to satisfy, either fully or partially, the following four properties: the adherence to the robot’s motion constraints, conformity with environmental constraints, completeness of the solution, and optimality of the generated trajectory. Achieving these properties typically requires a balance between planning quality and computational efficiency, as improving one aspect often comes at the expense of the other [6].

Common path-planning algorithms typically operate on binary maps, where environmental constraints are simplified to passable or non-passable regions, and the mobile robot is treated as a point mass [7,8,9,10]. These approaches perform well in structured environments such as warehouses and logistics facilities. However, in real-world scenarios, three-dimensional terrain significantly influences both the traversability and the cost of robot motion [11]. As a result, optimizing paths based solely on two-dimensional projections proves insufficient and often inaccurate.

Three-dimensional path planning methods are commonly employed in UAV applications, where 3D cells or polygonal representations are used to simplify the planning process [12,13]. Nevertheless, because UAVs can freely maneuver along the vertical axis, the assumptions underlying their planning algorithms differ substantially from those of ground mobile robots. As a result, UAV-oriented methods cannot be directly applied to terrestrial robots operating under strict kinematic and terrain constraints.

While concepts such as phase windows, coarse guidance, and Dubins-based regions have been widely applied in path planning, these techniques are often limited to static environments or initial coarse path generation. Existing methods, such as A* and RRT, utilize these components to refine paths, but they fail to fully account for real-time environmental dynamics, kinematic constraints, or dynamic terrain variations. This approach addresses these gaps by integrating dynamic phase windows and real-time feedback mechanisms, allowing for continuous refinement of paths based on environmental feedback and robot kinematics.

The posture of ground-operating mobile robots varies with the undulations of the terrain. In unstructured environments, in particular, posture variations can significantly affect the robot’s operational capability and stability [14]. To ensure safe and reliable autonomous operation, path planning for mobile robots must therefore incorporate comprehensive environmental and kinematic constraints. As a result, the cost function for path planning in unstructured environments should account not only for path length but also for factors such as terrain slope, vibration intensity, and the minimum turning radius of the robot.

To address these requirements, this paper proposes a novel dual-layer Hybrid-A* algorithm that integrates dynamic phase windows and operates on DEM elevation maps of unstructured environments. Unlike traditional methods that rely on static phase windows and coarse guidance, this approach introduces a dynamic phase-window adjustment strategy, enabling real-time path refinement based on environmental feedback and kinematic constraints. This dynamic adjustment ensures continuous optimization of paths, adapting to both environmental changes and robot motion limitations. The guidance layer provides landmark guidance points and a phase-window partitioning strategy, while the planning layer implements phase-window switching mechanisms and an enhanced Hybrid-A* method that accounts for both environmental and kinematic constraints.

1. A dual-layer Hybrid-A* path planning framework is developed to effectively reduce the search space size and improve overall path search efficiency.
2. A bicubic interpolation–based super-resolution reconstruction method is introduced to refine elevation maps in unstructured environments, providing a reliable basis for mobile robot posture estimation in the planning layer.
3. Phase windows constructed in the guidance layer, along with their associated heuristic cost functions, efficiently constrain the search region and guide the search direction.
4. An improved Hybrid-A* algorithm is designed in the planning layer, incorporating multi-dimensional cost functions to satisfy motion constraints, an adaptive expansion radius to enhance search-tree flexibility, and pruning and phase-window activation strategies to further improve search efficiency.

The structure of this paper is outlined as follows: Section 2 presents a review of the related work. Section 3 details the materials and methods employed in this study. Section 4 reports the experimental results and corresponding analysis. Section 5 summarizes the key contributions of this work and outlines promising directions for future research.

## 2. Related Work

Path planning is fundamentally a combinatorial optimization problem that seeks an optimal solution from all feasible paths satisfying specified constraints. Early studies on path planning mainly focused on finding paths within simplified models, such as sampling-based planning algorithms and graph search methods. However, the paths produced under such simplified assumptions often overlook the practical constraints encountered by mobile robots, leading to considerable discrepancies when applied in real-world scenarios. As a result, incorporating necessary environmental, kinematic, and dynamic constraints into path planning tasks across different application domains has become an important direction of current research.

Connecting the simplest coordinate sequences with straight lines often results in zigzag trajectories, causing mobile robots to frequently change motion states, leading to higher energy consumption and increased wear on motion mechanisms. In contrast, smooth path planning is better suited to the motion constraints of mobile robots. Qi et al. [15] highlight the importance of integrating heterogeneous sensors, such as wearable and environmental sensors, for improving the adaptability and accuracy of AIoT-based HAR systems, ensuring better recognition and decision-making in complex environments. One approach is to employ spline curves to smooth trajectories based on discrete waypoints generated by traditional planning algorithms. Jleilaty et al. [16] proposed a distributed control system that enhances the real-time performance and accuracy of path smoothing, addressing the challenges posed by high-curvature trajectories and collisions when waypoints are near obstacles.

Elbanhawi et al. proposed a continuous path smoothing method based on B-spline curves to address continuity and maximum curvature constraints imposed by non-holonomic motion in car-like robots [17]. Blažeč et al. introduced a minimum-time optimized path-planning algorithm using low-order Bézier curves to solve motion planning problems for automated guided vehicles (AGVs) in warehouse environments, particularly considering speed and acceleration constraints [18]. Although such smoothing techniques effectively control path continuity and curvature, they rely heavily on the quality of the initial discrete waypoints. When these waypoints lie close to obstacles, the smoothing process may produce excessively high-curvature curves or lead to collisions.

A second approach integrates discrete search with continuous state transitions to build continuous curve-based search trees, such as the Hybrid-A* algorithm [19] and the TargetTree-RRT* algorithm [20]. This strategy incorporates environmental and motion constraints directly into the search process. However, due to the rapid growth of nodes in the search tree, these methods are typically limited to comparatively small environments.

A third approach interprets discrete waypoints from conventional algorithms as sequential local path-planning subtasks, enabling progressive exploration across multiple simplified environmental models. For example, a global dynamic path planning method combining an improved A* algorithm with the Dynamic Window Approach (DWA) was developed for optimal path generation and obstacle avoidance in complex AGV environments [21]. Similarly, Ndidiamaka et al. proposed a hybrid model that integrates Rapidly-exploring Random Tree (RRT) and DWA with a pre-trained YOLOv7 object detection model to enhance obstacle detection and avoidance within the ROS framework [22]. In addition, Wang proposed a fuzzy logic path planning method based on geometric landmarks and motion constraints, combining the Dijkstra algorithm and fuzzy logic for mobile robot navigation in complex 2D terrains [23]. However, this sequential search mechanism may still result in substantial curvature variations when switching between intermediate target points, thus limiting its effectiveness in dynamic environments.

Path planning in three-dimensional environments is more suitable for complex, unstructured terrains, as it typically requires explicit 3D spatial representations, suitable constraint models for mobile robots, and comprehensive cost function formulations. Common 3D path-planning algorithms are commonly used in the field of unmanned aerial vehicles (UAVs). Afonso et al. proposed a traversability analysis and path planning technique based on mechanical effort to address autonomous navigation for unmanned ground vehicles operating in forest environments [24]. Their method extracts terrain gradient information and performs obstacle detection from 3D point cloud data, generating efficient paths avoiding unnecessary slope changes, potentially improving fuel economy, reducing equipment wear, and lowering operational risks. However, while UAVs can freely move along the vertical axis, ground mobile robots must account for both positional relationships and posture stability.

Hu et al. presented an integrated path-planning system for unmanned differential-steering vehicles in off-road environments. The system utilizes 3D terrain information, obstacle potential field functions, and nonlinear optimal control strategies to efficiently generate safe and dynamically feasible trajectories [25]. Similarly, Wu et al. proposed a DEM-AIA path planning framework that integrates Digital Elevation Models with an asymmetric inclination-aware strategy based on an improved A* algorithm, enabling pitch and roll angle estimation for each newly generated triangular cell [26]. This planner also provides feasible speed references along the path, enabling efficient navigation of off-road vehicles in complex terrain.

Han et al. [27] introduced an enhanced adaptive 3D path-planning algorithm (MS-W-Theta*) for mobile robots. Incorporating obstacle buffering techniques and an improved Theta* algorithm with minimum-snap trajectory smoothing, their method optimizes path planning in real-world applications, significantly enhancing path smoothness and efficiency.

Mobile robots operating in unstructured environments must simultaneously address the combined effects of environmental constraints and kinematic limitations. Moreover, the curse of dimensionality in large-scale environments significantly increases the difficulty of path searching and the computational burden of planning. To effectively mitigate trajectory accumulation errors in large-scale unstructured scenarios and reduce the complexity of tracking control in high-dimensional configuration spaces, a dual-layer Hybrid A* path-planning algorithm based on phase windows is proposed.

## 3. Materials and Methods

### 3.1. Experimental Platform

**Figure 1:**
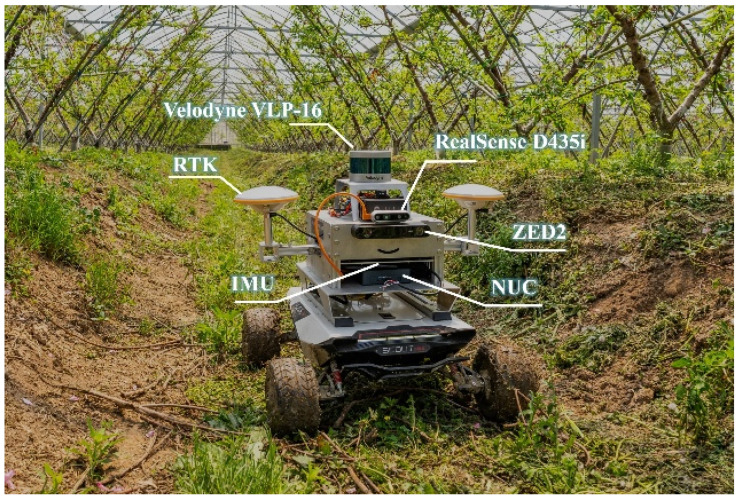
Experimental platform with integrated sensors.

The experimental platform, shown in Figure 1, is an agricultural robot integrated with various sensors, such as a depth camera (D435i, Intel RealSense, Intel Corporation, Santa Clara, CA, USA), a stereo camera (ZED2, Stereolabs, San Francisco, CA, USA), a LiDAR (VLP-16, Velodyne Lidar, San Jose, CA, USA), an inertial measurement unit (SI3200G MEMS, MeiTai, Shenzhen, China), and a GNSS receiver (UM482, Unicore Communications, Beijing, China). The onboard computing and processing unit is a NUC9VXQNX workstation (Intel Corporation, Santa Clara, CA, USA; Ubuntu 20.04.6 LTS; Intel E-2286M CPU @2.4–5.0 GHz; 32 GB RAM). The ground truth for localization is provided by the NovAtel SPAN-CPT system (NovAtel Inc., Calgary, AB, Canada), an enhanced RTK+INS navigation solution. The system development is based on the Robot Operating System (ROS) Noetic and C++. The robot utilizes a four-wheel differential-drive system that enables precise control of the turning radius and navigation through varying wheel speeds. Under the operating conditions considered in this study, the minimum turning radius of the platform is approximately 0.38 m, and this value is used as the turning-radius constraint in the path-planning algorithm.

### 3.2. Dual-Layer Environment Model

In this study, a three-fold upsampling approach is introduced to improve the resolution of the DEM, which is essential for fine-grained terrain modeling required for real-time robot operation. This method significantly enhances the accuracy of terrain features, like slope estimation and robot posture prediction, which are critical for path planning. Unlike conventional upsampling techniques that can lead to computational inefficiency or loss of accuracy, this approach strikes a balance between enhancing terrain detail and maintaining computational efficiency, ensuring real-time performance. The upsampling technique is integrated with bicubic interpolation to smooth high-frequency noise, providing a more accurate terrain representation for path planning.

#### 3.2.1. The Digital Elevation Model (DEM)

A DEM is a digital three-dimensional representation of surface elevations, commonly derived from sources like satellite imagery, LiDAR sensors, or topographic surveys. It is typically stored in a regular grid structure, where each grid point corresponds to an elevation value. DEMs provide an intuitive description of terrain variations and enable detailed analysis and computation of terrain features, such as slopes, ridges, and valleys. These models are essential for predicting and planning the posture changes experienced by mobile robots along their planned paths.

The source of the DEM data in this study is field-collected point clouds using a Velodyne VLP-16 LiDAR sensor (Velodyne Lidar, San Jose, CA, USA). The native resolution of the DEM is 0.6 m per pixel, chosen to balance computational efficiency and terrain detail. Higher resolution DEMs provide more accurate representations of the terrain, but they also result in higher computational costs. A 3× bilinear interpolation upsampling is further applied to enhance the DEM resolution to 0.2 m per pixel, matching the horizontal point spacing of the VLP-16 LiDAR (≈0.17 m) and ensuring the accuracy of slope calculation for path planning.

This trade-off between terrain detail and computational efficiency is a key aspect of the approach. Although higher resolution DEMs provide more accurate terrain representations, they also pose computational challenges, particularly in large-scale environments. To address this, a three-fold upsampling method enhances DEM resolution while maintaining computational feasibility for real-time robotic path planning. This balance is critical for ensuring that the path-planning algorithm remains efficient without sacrificing the accuracy of terrain features, such as slopes and ridges, which are vital for robot stability and navigation.

In this study, the DEM is processed with a three-fold upsampling approach to improve the resolution for path planning. This upsampling technique increases the spatial resolution of the DEM by a factor of three, allowing for finer terrain details while maintaining the computational efficiency needed for real-time operation. The rationale behind this upsampling approach is to improve the accuracy of terrain features, such as slope estimation, which are critical for path planning, without incurring excessive computational overhead.

The DEM is mathematically expressed as a two-dimensional array or matrix:

(1) $$\begin{array}{c} DEM=\left\{z_{ij}|i=1,2,\ldots ,n;\;j=1,2,\ldots ,m\right\}, \end{array}$$

where $z_{ij}$ represents the elevation value at position $\left(x_{i},y_{j}\right)$. The parameters *n* and *m* represent the number of rows and columns of the matrix, respectively, defining the DEM’s spatial size. The upsampling process creates a finer resolution grid, which is then used to calculate the robot’s path more accurately.

To ensure the quality of the DEM, potential noise characteristics arising from sensor errors or data acquisition limitations are considered. Simple smoothing filters, although effective at reducing noise, can blur fine terrain details, making them unsuitable for precise path planning. In contrast, this approach utilizes bicubic interpolation, which provides higher-order smoothing, effectively minimizing high-frequency noise while preserving critical terrain features. This allows for a more accurate terrain representation, especially in areas where features such as slopes and ridges significantly impact robot path planning.

A three-fold upsampling approach was chosen to improve the resolution of the DEM for path planning. The rationale for this approach is twofold: first, it provides finer terrain details, such as more accurate slope estimates, which are critical for safe robot navigation in unstructured environments; second, this upsampling method enhances DEM resolution without significantly increasing computational demands. The three-fold increase in resolution strikes a balance between improving terrain representation and maintaining the computational efficiency required for real-time path planning. This approach ensures that the path-planning algorithm processes the enhanced DEM data efficiently while capturing key terrain features.

#### 3.2.2. Construction of the Dual-Layer Environment Model

In three-dimensional path planning, DEMs are typically constructed such that each grid cell is larger than the robot to ensure smooth navigation. However, this design does not account for changes in the robot’s posture during movement. To address the challenge of posture estimation for mobile robots operating in unstructured terrains, this paper proposes a two-layer environmental model. The model supports the generation of guiding points in the guide layer and the execution of path searching in the planning layer. The guide layer rapidly generates guiding points using the original DEM and establishes phase windows for subsequent planning.

In bicubic interpolation, the value of each data point is influenced not only by its immediate neighboring points but also by the rate of change of adjacent samples, providing a more accurate representation of underlying trends. Compared to linear interpolation, which only considers straight-line relationships, bicubic interpolation captures more complex surface variations. This makes it particularly suitable for refining elevation maps in unstructured terrains, effectively eliminating jagged boundaries while producing smoother and more natural surfaces. Therefore, the proposed algorithm employs bicubic interpolation to construct high-resolution elevation maps for accurate posture estimation of mobile robots in the planning layer.

The bicubic interpolation function at the coordinates (x,y) is given in Equation (2).

(2) $$\begin{array}{c} z\left(x,y\right)=\sum_{i=N}^{3}\sum_{j=N}^{3}z\left(x_{i},y_{j}\right)\times W_{\left(x-x_{i}\right)}\times W_{\left(y-y_{j}\right)} \end{array}$$

Here, z(x,y) represents the elevation value at the point (x,y). The weight functions $W_{\left(x-x_{i}\right)}$ and $W_{\left(y-y_{j}\right)}$ are defined using the cubic convolution kernel proposed in [28], which achieves a balance between edge sharpness and surface smoothness through piecewise polynomial constraints.

**Figure 2:**
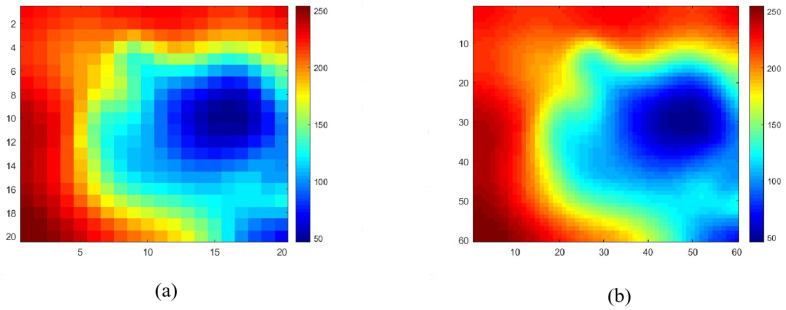
Comparison of the original low-resolution elevation map in (**a**) and the reconstructed high-resolution elevation map in (**b**).

Figure 2 illustrates the elevation maps before and after resolution enhancement, where subfigure (a) shows the original low-resolution elevation map, and subfigure (b) presents the high-resolution map obtained through bicubic interpolation. The reconstructed elevation map improves the spatial resolution by a factor of three compared to the original.

### 3.3. Guideline Layer Establishment Method

The guiding layer is responsible for generating waypoints and defining phase windows. Its primary purpose is to provide expansion directions for the search tree in the planning layer and reduce the effective search space, thereby enhancing the algorithm’s overall performance. The guiding layer workflow is as follows. First, the 3D A* algorithm is employed to obtain a sequence of guiding waypoints from the elevation map. Next, phase windows are established based on these waypoints. Finally, the departure cost within each phase window is assigned.

#### 3.3.1. Searching for Guide Waypoints

The A* algorithm, due to its optimality and computational efficiency, has become one of the most widely used graph-based path planning methods. In this study, the traditional A* algorithm is extended to a 3D multi-cost environment to obtain guiding landmarks in the guidance-layer map. The multiple cost components include distance, slope, and unknown threat. The distance cost represents the total travel distance of the mobile robot, the slope cost reflects the effect of pitch or roll during motion, and the unknown threat cost accounts for penalties incurred when traversing regions with undetectable hazards. The cost function of the improved 3D A* algorithm is expressed as follows:

(3) $$\left\{\begin{array}{c} {\hat{f}}_{\left(n\right)}=g_{\left(n\right)}+{\hat{h}}_{\left(n\right)}\\ g_{\left(n\right)}=\left(\omega_{thr}+\omega_{p}+1\right)d+g_{\left(n-1\right)}\\ \omega_{p}=\frac{\Delta z}{\sqrt{{\left(x_{n}-x_{n-1}\right)}^{2}+{\left(y_{n}-y_{n-1}\right)}^{2}+{\left(z_{n}-z_{n-1}\right)}^{2}}} \end{array}\right.$$

Here, $\left(\left(x_{n},y_{n},z_{n}\right)\right)$ denotes the current search position with its elevation, while $\left(\left(x_{n-1},y_{n-1},z_{n-1}\right)\right)$ represents the position and elevation from the previous step. The term $\omega_{p}$ is the slope factor weight. The values $g_{\left(n\right)}$ and $g_{\left(n-1\right)}$ represent the approach costs at the current and previous search locations, respectively. The term $\hat{h}\left(n\right)$ represents the estimated departure cost, calculated using the two-dimensional Euclidean distance between the current point and the goal point. Finally, $\hat{f}\left(n\right)$ represents the estimated total cost at the current search location.

The threat weight ($\omega_{thr}$) represents areas that may affect the robot’s operation, such as ponds, swamps, fires, and other hazardous regions. This weight can be adjusted based on the operating environment characteristics. In this algorithm, the threat weight is determined by the spatial distribution of multiple environmental hazards. Each hazard source located at position $p_{t_{i}}$ affects the threat level at point p using a spatial decay function. The cumulative threat weight is expressed as:

(4) $$\omega_{thr}\left(p\right)=\sum_{i=1}^{N}\omega_{max,i}\cdot \exp\left(-\frac{∥p-p_{t_{i}}{∥}^{2}}{2\sigma_{i}^{2}}\right)$$

where $\omega_{max,i}$ is the maximum threat weight for the *i*-th hazard, and $\sigma_{i}$ controls its spatial influence.

Guide markers are extracted at points where the direction changes along the path. Markers are classified into two categories: sparse for long, relatively straight segments, and dense for curved segments. The marker type is determined based on the distance between markers. Considering the motion characteristics of mobile robots, if the distance between markers is less than twice the minimum turning radius, they are labeled as dense; otherwise, they are labeled as sparse.

#### 3.3.2. Phase Windows

This section introduces a novel phase-window approach that combines guiding markers with Dubins curves to generate discretized, non-standard window shapes, referred to as phase windows, while similar approaches have been used in previous studies, such as coarse-to-fine path planning using fixed windows or Dubins-based regions for smoothing, this method offers dynamic adaptation by integrating real-time adjustments of window size and orientation based on environmental feedback and the robot’s kinematic constraints.

In traditional methods, fixed phase windows often lead to inefficient or infeasible paths in complex or dynamic environments due to rigidity in window placement and size. On the other hand, this approach introduces sparse guiding markers, strategically placed to adjust the window configuration dynamically, ensuring more flexible and real-time path refinement. The dynamic adaptation of the phase windows enables the robot to respond to changing terrain and obstacles, providing a more robust solution to path planning in unstructured environments.

Furthermore, the use of the Dubins curve to define feasible regions provides an efficient way to incorporate the minimum turning radius constraint into phase window construction. This allows the robot to accurately model its motion capabilities while avoiding excessive path curvature or infeasibilities, often seen in traditional fixed-window methods.

Sparse guiding markers are distributed at relatively large intervals with minimal variation in direction. This work proposes a novel phase-window construction method based on the Dubins curve [29], while the Dubins curve is commonly used to generate feasible paths for mobile robots by connecting two points with a specified curvature, this method introduces dynamic adjustment of the guiding markers based on real-time feedback and robot posture. This enables the phase windows to adapt to terrain variations and robot constraints, improving path accuracy and robustness in unstructured environments.

A Dubins curve produces a multi-segment trajectory connecting two coordinate points, given a specified curvature. Thus, it defines a feasible path for the robot to follow the guiding markers while maintaining the minimum turning radius. The region enclosed by the polyline formed by the Dubins curve and markers can be regarded as the search space that satisfies the robot’s turning-radius constraints.

Mathematically, the boundary of the phase window, denoted as W, is expressed as:

(5) $$W=\left\{\left(x,y\right)|\left(x,y\right)\in C_{\mathrm{Dubins}}\cup C_{\mathrm{Markers}}\right\},$$

where $C_{\mathrm{Dubins}}$ is the region defined by the Dubins curve, and $C_{\mathrm{Markers}}$ represents the area enclosed by the guiding markers. The robot is constrained to move within this region, ensuring that its motion adheres to the minimum turning radius constraint.

The procedure for constructing dynamic phase windows with sparse guiding markers is summarized in Algorithm 1. Each phase window is generated sequentially, with local starting points assigned based on the order of the sparse guiding markers. The subsequent guiding markers are used as endpoints for generating the phase windows via Dubins curves. In traditional methods, phase windows are fixed and do not adapt to environmental changes, resulting in frequent window switching. By contrast, this approach introduces dynamic window resizing based on terrain features, ensuring smooth transitions and reducing computational overhead.
**Algorithm 1** Procedure for generating phase window search areas guided by sparse markers.  1:**Input:** Array of key points key_points  2:**Output:** Recorded search areas on the map  3:num_points←length(key_points)  4:order_of_points←getOrder(key_points)  5:**for** i←1 **to** num_points **do**  6:    local_start_point ← key_points[*i*]  7:    **for** j←i+1 **to** num_points **do**  8:        end_point ← key_points[*j*]  9:        dubins_curve←createDubinsCurve(local_start_point,end_point)10:        search_area←encloseArea(dubins_curve,original_guide_path)11:        **Record** end_point as the guide point12:        guide_point←end_point13:        updateGuidePoint(guide_point)14:        **if** terminationConditionMet(search_area,dubins_curve) **then**15:           recordSearchArea(search_area)16:           **break**17:        **end if**18:    **end for**19:**end for**

The switching condition for the phase window is determined based on the following criteria:

1. Slope Variation: If the slope variation Δθ between successive guiding markers exceeds a threshold $\theta_{\max}$, the phase window switches. This condition can be expressed as:

(6) $$\Delta \theta =\left|\theta_{\mathrm{current}}-\theta_{\mathrm{previous}}\right|>\theta_{\max}.$$

2. Environmental Threats: If an obstacle or environmental threat $O_{\mathrm{threat}}$ is detected within a critical distance $d_{\mathrm{threat}}$, the phase window will switch. This condition can be expressed as:

(7) $$d_{\mathrm{threat}}<d_{\mathrm{critical}},$$

where $d_{\mathrm{critical}}$ is a predefined threshold distance indicating proximity to an obstacle.

3. Excessive Window Size: If the phase window exceeds a predefined size $W_{\max}$, the window switches to ensure that it remains manageable for path planning:

(8) $$W_{\mathrm{size}}>W_{\max},$$

where $W_{\mathrm{size}}$ is the current size of the phase window.

The construction of a phase window terminates when any of the following conditions are met:

1. Significant Slope Variation: If the slope variation between the current and previous guiding markers exceeds a threshold $\Delta \theta_{\max}$, the window construction is terminated. The condition can be expressed as:

(9) $$\Delta \theta >\Delta \theta_{\max}.$$

2. Environmental Threats: If an obstacle or threat is detected within a critical distance $d_{\mathrm{critical}}$, the phase window construction terminates to avoid collisions:

(10) $$d_{\mathrm{threat}}<d_{\mathrm{critical}}.$$

3. Excessive Window Size: If the window size exceeds the maximum allowed size $W_{\max}$, the window construction is stopped:

(11) $$W_{\mathrm{size}}>W_{\max}.$$

**Figure 3:**
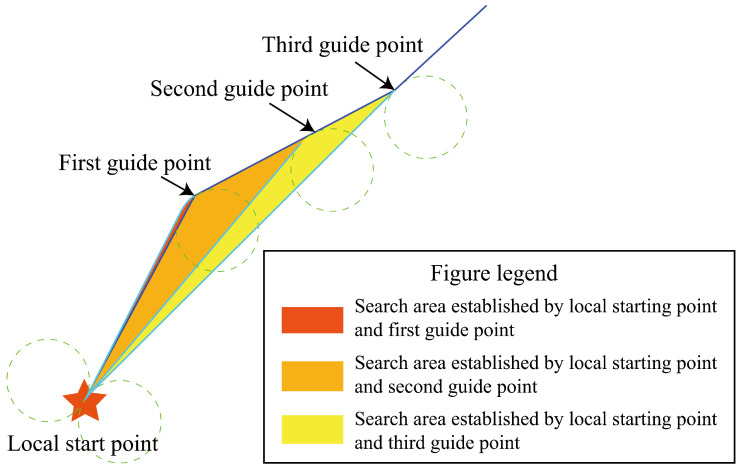
Visualization of phase window search areas guided by sparse markers.

Once any of these conditions are met, the system proceeds to generate the next phase window, ensuring that the path remains feasible and efficient (Figure 3).

#### 3.3.3. Heuristic Cost in Phase Windows

The heuristic cost guides the path-planning algorithm in the planning layer. The guiding markers constructed here store the accumulated cost from the starting point to each marker. The heuristic cost at each guiding marker is obtained by computing the difference between the endpoint cost and the marker cost. The heuristic cost values for other positions in a phase window are then computed based on these guiding markers.

(a) Heuristic cost in phase windows guided by sparse markers

The heuristic cost $h(x_{d},y_{d})$ at a coordinate $\left(x_{d},y_{d}\right)$ within a phase window constructed using sparse guiding markers is calculated using Equation (12).

(12) $$\left\{\begin{array}{c} \hat{h}\left(x_{d},y_{d}\right)=\hat{h}\left(x_{ls},y_{ls}\right)\frac{DIS_{gp}}{DIS_{ls}+DIS_{gp}}+\hat{h}\left(x_{gp},y_{gp}\right)\frac{DIS_{ls}}{DIS_{ls}+DIS_{gp}}\\ DIS_{gp}=\sqrt{{\left(x_{gp}-x_{d}\right)}^{2}+{\left(y_{gp}-y_{d}\right)}^{2}}\\ DIS_{ls}=\sqrt{{\left(x_{d}-x_{ls}\right)}^{2}+{\left(y_{d}-y_{ls}\right)}^{2}} \end{array}\right.$$

$g_{\left({\underline{x}}_{\left(n\right)}\right)}=g_{\left({\underline{x}}_{\left(n-1\right)}\right)}+\sum_{i=2}^{m}\left[\frac{1}{\omega_{\mathrm{grad}}+\epsilon }+\frac{1}{\omega_{\mathrm{bump}}+\epsilon }+\frac{1}{\omega_{\mathrm{thr}}+\epsilon }\right]g_{\mathrm{dis}}$

Here, $\left(x_{ls},y_{ls}\right)$ denotes the starting marker of the phase window, and $\left(x_{gp},y_{gp}\right)$ denotes the ending guiding marker. When phase windows overlap, the smaller heuristic cost is recorded. This ensures the heuristic cost consistently decreases toward the global endpoint.

(b) Heuristic cost in phase windows guided by dense markers

**Figure 4:**
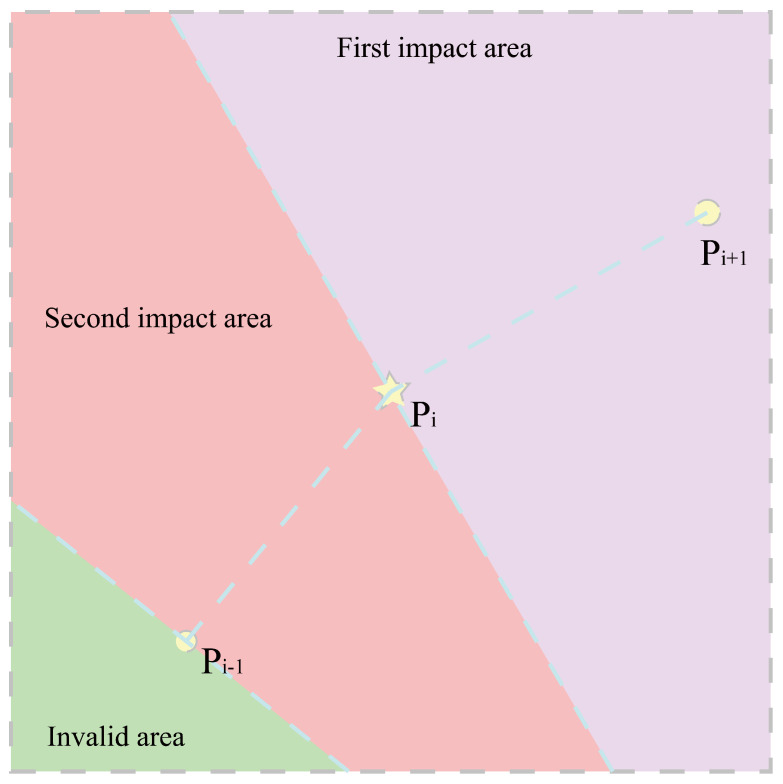
Heuristic cost in phase windows guided by dense markers.

The phase window guided by dense markers contains three points $\left(P_{i-1},P_{i},P_{i+1}\right)$. By projecting $P_{i-1}$ and $P_{i}$ perpendicularly onto vectors $\vec{P_{i-1}P_{i}}$ and $\vec{P_{i}P_{i+1}}$, the phase window is divided into three subregions (as illustrated in Figure 4). Coordinates falling within the invalid region are excluded from the heuristic cost computation. For the remaining two valid regions, the heuristic cost is computed using Equation (12). In the first region, $P_{i}$ is the starting marker and $P_{i+1}$ the ending marker. In the second region, $P_{i-1}$ is the starting marker and $P_{i}$ the ending marker.

(c) Heuristic cost in special phase windows

At the starting guiding marker, besides the phase window region defined by sparse markers, the heuristic cost in the supplementary square region is the sum of the start point’s heuristic cost and the Euclidean distance from the start point to the coordinate. At the ending guiding marker, except where heuristic costs are already assigned in other phase windows, the heuristic cost in the supplementary square region is defined as the Euclidean distance from the coordinate to the endpoint. Thus, the search direction in the planning layer always guides the robot toward the global endpoint, as summarized in Algorithm 2.
**Algorithm 2** Heuristic cost calculation in phase windows.  1:**Input:** Guide markers and coordinate $\left(x_{d},y_{d}\right)$  2:**Output:** Heuristic cost $h(x_{d},y_{d})$  3:**if** phase window guided by sparse markers **then**  4:    Compute $DIS_{gp}$ and $DIS_{ls}$  5:    Compute $h(x_{d},y_{d})$ using the weighted-average formula (Equation (12))  6:    **if** overlap exists between phase windows **then**  7:        Record the minimum value of $h(x_{d},y_{d})$  8:    **end if**  9:**end if**10:**if** phase window guided by dense markers **then**11:    Partition the phase window into subregions12:    **if** $\left(x_{d},y_{d}\right)$ lies in a valid subregion **then**13:        Compute $h(x_{d},y_{d})$ based on the corresponding guide markers14:    **end if**15:**end if**16:**if** special phase window at the start or end marker **then**17:    **if** $\left(x_{d},y_{d}\right)$ is at the start-marker region **then**18:        $h\left(x_{d},y_{d}\right)=h_{\mathrm{start}}+\mathrm{EuclideanDistance}$19:    **else if** $\left(x_{d},y_{d}\right)$ is at the end-marker region **then**20:        $h(x_{d},y_{d})=\mathrm{EuclideanDistance}\;\mathrm{to}\;\mathrm{endpoint}$21:    **end if**22:**end if**23:**Return** $h(x_{d},y_{d})$

### 3.4. Planning Layer Establishment Method

The planning layer integrates an enhanced Hybrid-A* algorithm that performs path planning within the phase windows generated by the guidance layer, ensuring compliance with mobile robot motion constraints. First, the algorithm introduces an improved cost function adaptable to complex, unstructured environments. Second, it incorporates an adaptive turning-radius selection mechanism to prevent unnecessary turns or loops caused by a fixed turning radius. Finally, a pruning strategy is employed to accelerate the path search and reduce computational overhead.

#### 3.4.1. Cost Function

The cost function of the improved Hybrid-A* algorithm is given in Equation (13).

(13) $$\left\{\begin{array}{c} {\hat{f}}_{\left({\underline{x}}_{\left(n\right)}\right)}=g_{\left({\underline{x}}_{\left(n\right)}\right)}+{\hat{h}}_{\left({\underline{x}}_{\left(n\right)}\right)}\\ g_{\left({\underline{x}}_{\left(n\right)}\right)}=g_{\left({\underline{x}}_{\left(n-1\right)}\right)}+\sum_{i=2}^{m}\left(1+\frac{\omega_{grad}}{\omega_{grad,\max}+\epsilon }+\frac{\omega_{bump}}{\omega_{bump,\max}+\epsilon }+\frac{\omega_{thr}}{\omega_{thr,\max}+\epsilon }\right)g_{dis} \end{array}\right.$$

In this formulation, the heuristic cost $\hat{h}\left(xn\right)$ represents the estimated cost to reach the endpoint, whereas the approach cost $g_{\left(x_{n}\right)}$ denotes the accumulated cost from start to current position. The term $g_{dis}$ denotes the distance cost for each subdivided segment of the expansion tree, and *m* is the number of subdivisions per segment. The gradient weight $\omega_{grad}$ represents the inclination of the robot on the terrain; the bumpiness weight $\omega_{bump}$ quantifies the robot’s vibration intensity; and the threat weight $\omega_{thr}$ reflects the potential risk associated with unknown environmental hazards.

To ensure the stability and generalizability of the cost function, each weight term is normalized by dividing it by its maximum value (e.g., $\omega_{grad,\max}$) plus a small constant ϵ to prevent division by zero. Normalization standardizes the range of values for $\omega_{grad}$, $\omega_{bump}$, and $\omega_{thr}$, ensuring that no single term dominates the total cost due to differences in their physical units or magnitudes.

(a) Distance cost $\left(g_{dis}\right)$

The distance cost represents the actual traveling distance and is computed as the three-dimensional Euclidean distance between two adjacent points:

(14) $$g_{dis}=\sqrt{{\left(x_{i}-x_{i-1}\right)}^{2}+{\left(y_{i}-y_{i-1}\right)}^{2}+{\left(z_{i}-z_{i-1}\right)}^{2}}$$

(b) Gradient weight $\left(\omega_{grad}\right)$

The gradient weight is calculated based on the robot’s physical model and local terrain conditions, allowing the algorithm to evaluate the robot’s inclination at each position. Integrating the gradient weight into the cost function helps prevent slipping, tipping, and energy wastage on steep terrain.

(15) $$\left\{\begin{array}{c} \omega_{grad}=\tan\left(\max\left(\theta_{f-b},\theta_{l-r},\theta_{fl-br},\theta_{fr-bl}\right)\right)\\ \theta_{f-b}=\tan^{-1}\;\left(\frac{\frac{z_{fl}+z_{fr}}{2}-\frac{z_{bl}+z_{br}}{2}}{\sqrt{{\left(x_{fl}-x_{bl}\right)}^{2}+{\left(y_{fl}-y_{bl}\right)}^{2}}}\right)\\ \theta_{l-r}=\tan^{-1}\;\left(\frac{\frac{z_{fl}+z_{bl}}{2}-\frac{z_{fr}+z_{br}}{2}}{\sqrt{{\left(x_{fl}-x_{fr}\right)}^{2}+{\left(y_{fl}-y_{fr}\right)}^{2}}}\right)\\ \theta_{fl-br}=\tan^{-1}\;\left(\frac{z_{fl}-z_{br}}{\sqrt{{\left(x_{fl}-x_{br}\right)}^{2}+{\left(y_{fl}-y_{br}\right)}^{2}}}\right)\\ \theta_{fr-bl}=\tan^{-1}\;\left(\frac{z_{fr}-z_{bl}}{\sqrt{{\left(x_{fr}-x_{bl}\right)}^{2}+{\left(y_{fr}-y_{bl}\right)}^{2}}}\right) \end{array}\right.$$

The spatial coordinates of the four wheels of the mobile robot are defined as:

$$\left[\begin{array}{c} P_{\text{left-front-wheel}}\\ P_{\text{left-rear-wheel}}\\ P_{\text{right-front-wheel}}\\ P_{\text{right-rear-wheel}} \end{array}\right]=\left[\begin{array}{ccc} x_{fl} & y_{fl} & z_{fl}\\ x_{bl} & y_{bl} & z_{bl}\\ x_{fr} & y_{fr} & z_{fr}\\ x_{br} & y_{br} & z_{br} \end{array}\right]$$

(c) Bumpiness weight $\omega_{\mathrm{bump}}$

The bumpiness weight evaluates robot posture changes along the path segment. Incorporating this weight helps reduce mechanical wear and localization errors due to excessive vibration. To ensure stability and minimize abrupt variations, a smoothing strategy for the gradient differences is introduced. The bumpiness weight $\omega_{\mathrm{bump},i}$ is computed from the smoothed gradient differences between consecutive nodes, so larger values correspond to stronger posture fluctuations along the path.

(16) $$\left\{\begin{array}{c} \omega_{\mathrm{bump}}=\left\{\begin{array}{cc} \frac{N_{\mathrm{now}}}{N_{\max}}, & N_{\mathrm{now}}\leq N_{\max}\\ \alpha \cdot N_{\mathrm{now}}, & N_{\mathrm{now}}>N_{\max} \end{array}\right.\\ N_{\mathrm{now}}=\left(\omega_{\mathrm{grad},i}-\omega_{\mathrm{grad},i-1}\right)\cdot w_{\mathrm{smooth}}\\ N_{\max}=f\left(\mathrm{robot}’s\;\max\;\mathrm{slope},\;\mathrm{terrain}\;\mathrm{characteristics},\;\mathrm{or}\;\mathrm{experimental}\;\mathrm{data}\right) \end{array}\right.$$

Here, $\omega_{\mathrm{grad},i}$ denotes the gradient weight at the *i*-th node of the expansion tree, and $N_{\max}$ represents the maximum allowable change in gradient for safe robot operation. When the local gradient change exceeds $N_{\max}$, the penalty associated with the bumpiness term is saturated at its upper bound, so that excessively large variations do not lead to unbounded costs. The smoothing factor $w_{\mathrm{smooth}}$ helps reduce abrupt gradient variations by applying a weighted average of the gradient differences. This ensures that the bumpiness term remains stable and does not destabilize the search process.

#### 3.4.2. Adaptive Turning Radius

This section introduces two methods for selecting an adaptive turning radius. Unlike traditional fixed-radius search strategies, these methods allow more flexible determination of the search tree’s expansion direction based on guidance layer information.

(a) Guide point radius

**Figure 5:**
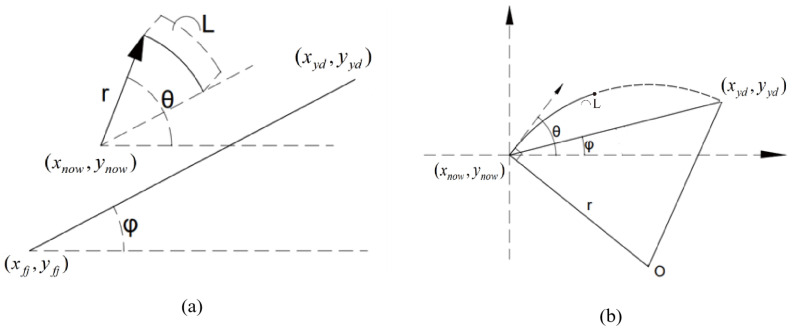
Illustration of the guide point radius in (**a**) and the guide direction radius in (**b**).

First, the method is called the guide point radius. A circular arc is constructed from the current search position $\left(x_{d},y_{d}\right)$ and heading direction $\left(\theta_{d}\right)$ toward the guide point $\left(x_{gp},y_{gp}\right)$. This arc is called the guide point arc, and its radius is termed the guide point radius. If the guide point radius exceeds the minimum turning radius of the mobile robot, the search tree expands in that direction (Figure 5). Equation (17) calculates the guide point radius.

(17) $$r_{gp}=\frac{\sqrt{{\left(x_{gp}-x_{d}\right)}^{2}+{\left(y_{gp}-y_{d}\right)}^{2}}}{2\sin\left(\tan^{-1}\;\left(\frac{y_{gp}-y_{d}}{x_{gp}-x_{d}}\right)-\theta_{d}\right)+\epsilon }$$

To prevent numerical instability when the heading aligns closely with the target direction, a small constant ϵ is added to the denominator. This ensures that the denominator never approaches zero, thus stabilizing the guide point radius computation.

(b) Guide direction radius

The direction from the local start point to its guide point is defined as the guide direction. The angle between the guide direction and the robot’s current heading is termed the adjustment angle. From the current position, an arc can be drawn using the adjustment angle as the central angle and the search step as the arc length. This arc is referred to as the guide direction arc, and its radius is the guide direction radius. If this radius exceeds the minimum turning radius of the mobile robot, the search tree is expanded accordingly (Figure 5). Equation (18) computes the guide direction radius.

(18) $$\left\{\begin{array}{c} r_{gd}=\frac{L}{\overset{⌢}{\theta -\psi }}\\ \tan\left(\psi \right)=\frac{y_{gp}-y_{ls}}{x_{gp}-x_{ls}} \end{array}\right.$$

Here, θ and ψ denote the current search direction and the guidance direction, respectively. *L* represents the search step size. $\left(x_{gp},y_{gp}\right)$ is the coordinate of the guide point, and $\left(x_{ls},y_{ls}\right)$ is the coordinate of the local start point.

#### 3.4.3. Pruning Method

The pruning method is designed to address two issues during path search: (1) the generation of unnecessary search branches that deviate from the expected search direction, and (2) the repeated generation of search nodes in locally dense regions, leading to redundant nodes with similar positions and headings. To mitigate these problems, the following pruning strategies are adopted:

1. Phase window constraint: Nodes outside the active phase window are pruned to restrict the search to dynamically defined feasible regions.
2. Directional consistency: Nodes forming an obtuse angle with direction ψ are removed to prevent reverse or divergent expansion of the search tree.
3. Redundancy suppression: New nodes are discarded if their distance from an existing node is smaller than the robot’s width and heading difference is $\Delta \theta <7.5^{\circ }$, reducing local redundancy.

#### 3.4.4. Activation Method for Phase Windows

Phase windows are a sequence of guided search regions generated by the guidance layer, each annotated with heuristic costs. In the planning layer, the expansion tree grows exponentially, which can severely reduce search efficiency in large-scale environments. To alleviate this, this section proposes an activation mechanism based on phase window activity levels, aiming to reduce candidate nodes during the search.

Assume that the global map contains *n* phase windows. Let $Win_{i}$ be the phase window containing the current node. Based on its index position, the state of each window is defined as follows:

(19) $$Windows_{j}\in \left\{\begin{array}{cc} Active, & i-10\leq j\leq n\\ Semi\text{-}active, & i-20\leq j<i-10\\ Inactive, & j<i-20 \end{array}\right.$$

During node expansion, the next node is selected with the lowest total cost from: (1) all nodes residing in active phase windows, and (2) 20% randomly selected nodes from semi-active phase windows. The pseudocode for the phase-window activation method is provided in Algorithm 3.
**Algorithm 3** Phase window activation method.  1:**Input:** Current node currentNode; phase window set *W*; current window index *i*; node list *N*  2:**Output:** Updated node list *N*  3:Initialize ActiveNodes ←∅, SemiActiveNodes←∅  4:**for** j←1 **to** length(W) **do**  5:    **if** i−10≤j≤i **then**  6:        Mark $W_{j}$ as Active  7:        **for all** node *n* in $W_{j}$ **do**  8:           Add *n* to ActiveNodes  9:        **end for**10:    **else if** i−20≤j<i−10 **then**11:        Mark $W_{j}$ as Semi-active12:        Select randomly 20% of nodes in $W_{j}$13:        **for all** selected nodes *n* **do**14:           Add *n* to SemiActiveNodes15:        **end for**16:    **else**17:        Mark $W_{j}$ as Inactive18:        Remove all nodes *n* in $W_{j}$ from *N*19:    **end if**20:**end for**21:N←ActiveNodes∪SemiActiveNodes22:**Return** *N*

## 4. Results

### 4.1. Simulation Experiment

The dual-layer environment modeling process reconstructs two resolution models representing the same environment. The lower-resolution model guides the construction of phase windows, resulting in multiple phase windows from start to endpoint, with decreasing heuristic costs recorded within each window. These phase windows are then mapped onto the high-resolution model, where a path satisfying the robot’s motion constraints is searched using the planning layer algorithm.

**Figure 6:**
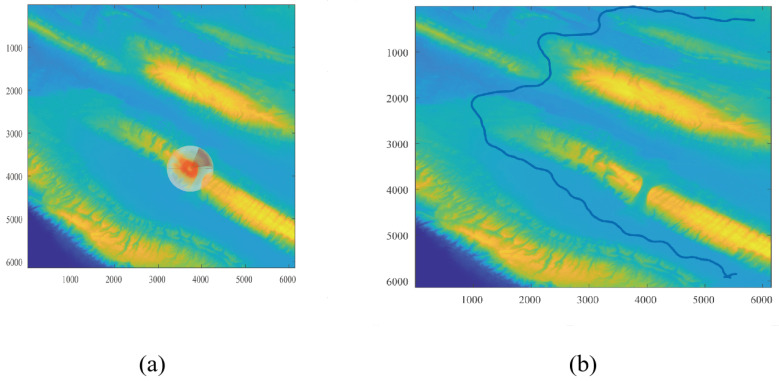
Illustration of the reconstructed environmental model of Map 2 in (**a**) and the planned route in Map 2 in (**b**).

**Figure 7:**
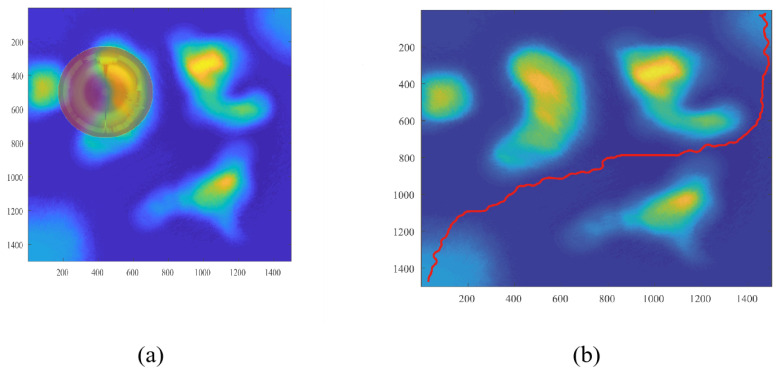
Illustration of the reconstructed environmental model of Map 1 in (**a**) and the planned route in Map 1 in (**b**).

Figure 6 illustrates this process on Map 2, where subfigure (a) shows the reconstructed low-resolution model used for generating phase windows, and subfigure (b) shows the final path searched on the corresponding high-resolution model. Figure 7 presents the same workflow on Map 3, where subfigure (a) shows the low-resolution reconstruction and generated phase windows, and subfigure (b) shows the resulting planned path on the high-resolution model.

As shown in these figures, the proposed algorithm effectively plans a path in a large unstructured environment that meets the robot’s motion constraints. The planned path avoids steep slopes and bumpy sections and satisfies the directional requirements of both start and end points, ensuring safe and feasible navigation across complex terrain.

**Figure 8:**
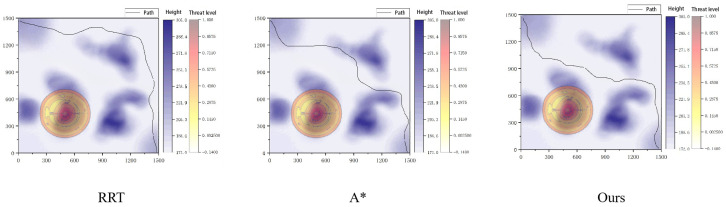
Visualization of the planned path on the reconstructed elevation map of Map 1 where the height distribution and threat level are jointly displayed to illustrate the terrain characteristics around the phase window center.

Figure 8 shows the planning process visualized on the reconstructed elevation map of Map 1, with both the elevation field and threat distribution. The concentric colored structure marks the phase window centered on the current expansion region, encoding heuristic costs from the low-resolution model. Mapped to the high-resolution elevation map, this window constrains the search direction and helps the planning layer avoid high-threat areas and unfavorable slopes. The resulting planned trajectory, shown in black, forms a smooth and feasible route across the unstructured environment, demonstrating that the proposed algorithm maintains stability in complex terrain and uses hierarchical environment representation for robust path planning.

Table 1 presents a comparative evaluation of the performance of four different path planning methods: A*, RRT, Hybrid A*, and the proposed method, using a Digital Elevation Model (DEM). The key metrics evaluated include path length, maximum slope, threat exposure, and planning time.

The proposed method generates the shortest path at 166.0 m, outperforming all other methods. The Hybrid A* method produces the next shortest path at 170.8 m, followed by A* (191.2 m) and RRT (211.8 m). Shorter paths reduce travel time and energy consumption, making them ideal for real-time robotic applications in challenging environments.

In terms of terrain steepness, the proposed method exhibits the lowest maximum slope of 15.5°, significantly lower than A* (24.7°) and RRT (22.5°). Lower slopes ensure better stability and safety in hazardous terrain.

The proposed method achieves the lowest threat exposure with a distance of 10.5 m, the best result among all methods. In contrast, A* and RRT have relatively higher threat exposure distances of 36.0 m and 29.2 m, respectively. The Hybrid A* method provides an intermediate threat exposure distance of 18.0 m, while DEM-based A* and other planners show similar results. Minimizing threat exposure is essential for reducing the robot’s risk in dangerous environments.

When considering computational efficiency, the proposed method excels with a planning time of 0.66 s, less than half of Hybrid A*’s time (1.50 s) and significantly faster than RRT (2.45 s). This fast planning time is crucial for real-time applications, ensuring the robot can quickly adapt to environmental changes.

Overall, the results confirm that the proposed method achieves the best performance across all metrics, providing the shortest path, lowest slope, minimal threat exposure, and fastest planning time. Improvements stem from the dual-layer architecture and terrain-aware cost model, which optimize safety and efficiency. The proposed method’s ability to enhance path planning quality while maintaining computational efficiency makes it an ideal choice for real-time robotic navigation in complex environments.

Table 2 summarizes the execution performance of different planning methods in a real outdoor DEM environment. The proposed method shows the best overall performance, with the lowest average position error (0.40 m) and maximum position error (0.80 m), as well as the shortest execution time (38.5 s) and the lowest average slope (7.2°). In comparison, A* and RRT exhibit larger position errors, longer execution times, and higher average slopes, showing that the proposed method offers superior accuracy, efficiency, and stability in real-world applications.

While A* and RRT achieve relatively shorter execution times than some other methods, they result in higher position errors, particularly in challenging terrain. The proposed method not only reduces execution time but also ensures more precise path tracking with the lowest slope, making it more suitable for real-world deployment, where terrain variability and execution accuracy are crucial. Additionally, DEM-based A* and Improved Theta variants offer competitive performance, but they still lag behind the proposed method in both accuracy and execution time. These results highlight the effectiveness of the proposed method in balancing path accuracy, execution time, and terrain adaptability for robotic navigation.

**Figure 9:**
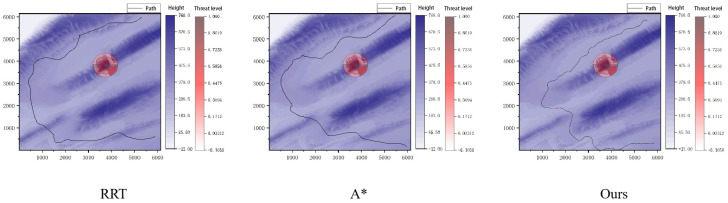
Visualization of the planned path on the reconstructed elevation map of Map 2 where the height distribution and threat level are jointly presented to show the terrain conditions around the phase window center.

Figure 9 shows the planning results on the reconstructed elevation map of Map 2, with height distribution and threat level displayed. The central colored region represents the phase window generated by the low-resolution model, with its position projected onto the high-resolution map. The gradient pattern in the blue colormap reflects terrain elevation, while the overlaid red tent map indicates locally aggregated threat information, guiding the planning layer’s expansion direction. The final planned path is drawn as a continuous black line, successfully bypassing high-threat regions and steep terrain changes, demonstrating that the proposed dual-layer method provides effective global guidance while ensuring local feasibility on the high-resolution map.

In contrast, the proposed dual-layer framework improves both efficiency and terrain adaptiveness. By integrating a coarse 3D A* guidance layer with phase-window–constrained Hybrid A* optimization, the method reduces planning time to 0.65 s, a 56.1% improvement over the single-layer baseline. The dual-layer structure also produces a slightly shorter path (168.0 m) and reduces the maximum slope from 15.8° to 15.0°, showing that the global guidance helps avoid steeper terrain without compromising path quality. Overall, these results clearly demonstrate that the dual-layer design significantly enhances both search efficiency and terrain safety in the planning process.

Table 3 compares the single-layer Hybrid A* planner with the proposed dual-layer framework. The single-layer approach requires 1.48 s and expands a larger search space, resulting in a maximum terrain slope of 15.8° along the path. The lack of global guidance leads the planner to explore more unnecessary nodes, increasing both computation time and the likelihood of traversing suboptimal terrain.

Table 4 shows the effect of removing terrain-related costs. Without the slope cost, the maximum slope increases to 20.5°, indicating that the planner tends to choose steeper and less safe terrain. Removing the bumpiness cost keeps slope and threat exposure low but leads to a less smooth trajectory, confirming that bumpiness cost is essential for improving terrain comfort. The absence of the threat cost causes the largest performance drop, with threat exposure increasing from 10.0 m to 21.8 m, more than doubling the distance traveled in hazardous regions. In contrast, the full-cost formulation achieves the lowest slope (15.0°) and minimum threat exposure (10.0 m), showing that all three cost terms contribute and complement each other to ensure a safer path.

**Figure 10:**
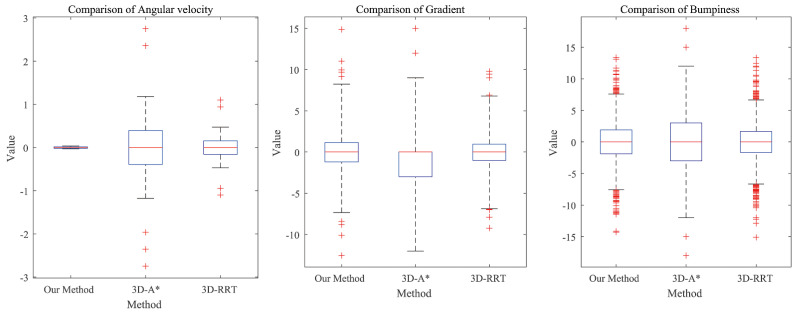
Algorithm performance box diagram.

To demonstrate the advantages of the proposed algorithm, comparative experiments were conducted in a 3D environment using the A* algorithm, the RRT algorithm, the potential field method, and a fuzzy logic path planner. The potential field method and the fuzzy logic planner exhibited poor performance in complex, unstructured environments, frequently getting trapped in local minima and failing to reach the goal. Therefore, the comparison primarily focuses on the A* and RRT algorithms, with which the paths generated by the proposed method are compared. The comparison considers angular velocity, terrain gradient, and bumpiness. The results of these comparisons are presented in Figure 10.

The proposed algorithm significantly reduces the heading angle change while maintaining excellent control over both terrain gradient and bumpiness. These improvements enhance path-tracking performance and overall safety.

### 4.2. Real-World Experiments

Real-world experiments were conducted on an integrated robotic platform. For accurate ground-truth localization, a NovAtel SPAN-CPT unit—providing enhanced RTK+INS navigation—was employed. System development and integration were completed in C++ within the ROS Noetic framework.

**Figure 11:**
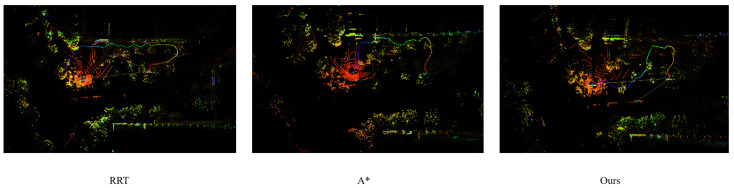
Visualization of the planned trajectory overlaid on the LiDAR point cloud environment where the trajectory is shown in color for visual contrast and the point cloud is rendered in grayscale and the color does not encode additional physical quantities.

To validate the planned trajectory in real scenarios, the final path is mapped onto the LiDAR-based point cloud of the experimental orchard, as shown in Figure 11. The point cloud preserves fine-grained geometric features such as vegetation distribution, ground roughness, and structural obstacles, which are not represented in the elevation map. Overlaying the planned route onto the 3D point cloud allows a thorough examination of the trajectory’s feasibility in real unstructured environments. The results show that the proposed algorithm successfully avoids dense vegetation clusters and maintains safe clearance from irregular terrain while preserving the heading requirements at both the start and end locations. This demonstrates that the planned path is not only theoretically feasible in the modeled elevation space but also practically executable in the real-world environment captured by the LiDAR sensor.

**Figure 12:**
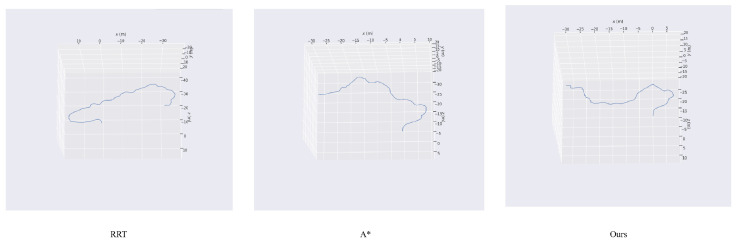
Visualization of the planned trajectory in the three dimensional elevation space.

The planned trajectory is further visualized in three-dimensional space to evaluate its geometric feasibility and smoothness in real terrain. As shown in Figure 12, the trajectory is projected onto the 3D elevation map, where variations in altitude, slope, and surface curvature are explicitly preserved. The visualized path shows that the algorithm generates a continuous and dynamically feasible trajectory that adapts to the terrain elevation. The path maintains stability over uneven terrain, avoids regions with steep gradients, and ensures smooth transitions between ascent and descent segments, which are essential for maintaining the robot’s posture and motion constraints during execution. This visualization confirms that the high-resolution results align with the environment’s three-dimensional structure.

## 5. Conclusions

This paper proposes a dual-layer Hybrid A* path-planning algorithm designed for unstructured environments. The algorithm effectively addresses the environmental and motion constraints encountered by mobile robots in complex, unstructured environments. First, a super-resolution reconstruction of the elevation map is applied to enable accurate estimation of the robot’s posture. Second, a dual-layer planning framework is proposed, in which the guidance layer constructs phase windows to steer the search direction and guide the expansion of the planning-layer search tree. Third, the planning layer incorporates multiple cost functions, an adaptive expansion radius, pruning strategies, and a phase-window activation mechanism. Experimental results demonstrate that the proposed algorithm successfully generates feasible paths that satisfy the robot’s motion constraints, including turning radius, posture stability, bumpiness, and potential environmental threats.

Future work will focus on active exploration strategies for unknown environments. The proposed algorithm can provide smoother trajectories, reducing disturbances from posture variations and sensor vibrations. Additionally, integrating aerial drones to collect environmental information and guide real-time path exploration for ground robots will also be explored.

## Figures and Tables

**Table 1:** Planning performance comparison on a DEM map.

Method	Path Length (m)	Max Slope (°)	Threat Exposure (m)	Planning Time (s)
A*	191.2	24.7	36.0	1.90
RRT	211.8	22.5	29.2	2.45
Proposed	166.0	15.5	10.5	0.66
DEM-based A*	175.3	18.1	20.9	1.50
Traversability planners	180.5	20.2	22.0	1.75
Improved Theta* variants	170.8	16.5	18.0	1.30

**Table 2:** Actual execution performances of different methods.

Method	Average Position Error (m)	Max Position Error (m)	Execution Time (s)	Average Slope (°)
A*	0.85	1.50	45.2	12.5
RRT	1.20	2.10	50.8	11.8
Proposed	0.40	0.80	38.5	7.2
DEM-based A*	0.70	1.30	42.0	9.0
Traversability planners	0.95	1.70	48.3	10.5
Improved Theta* variants	0.55	1.10	40.1	8.1

**Table 3:** Ablation study of the dual-layer planning framework on Map 1.

Configuration	Path Length (m)	Planning Time (s)	Max Slope (°)
Single-layer Hybrid A*	171.5	1.48	15.8
Dual-layer (proposed)	168.0	0.65	15.0
Improvement	−2.0%	−56.1%	−5.1%

**Table 4:** Ablation of terrain-related cost components.

Configuration	Max Slope (°)	Threat Exposure (m)	Path Length (m)
w/o slope cost	20.5	11.2	166.5
w/o bumpiness cost	16.2	10.5	167.3
w/o threat cost	16.0	21.8	165.9
Full cost (proposed)	15.0	10.0	168.0

## Data Availability

The original contributions presented in this study are included in the article. Further inquiries can be directed to the corresponding authors.

## References

[1] Su Q., Yu W., Liu J. Mobile robot path planning based on improved ant colony algorithm. Proceedings of the 2021 Asia-Pacific Conference on Communications Technology and Computer Science (ACCTCS).

[2] Chen G., You H., Huang Z., Fei J., Wang Y., Liu C. (2022). An Efficient Sampling-Based Path Planning for the Lunar Rover with Autonomous Target Seeking. Aerospace.

[3] Vagale A., Oucheikh R., Bye R.T., Osen O.L., Fossen T.I. (2021). Path planning and collision avoidance for autonomous surface vehicles I: A review. J. Mar. Sci. Technol..

[4] Filotheou A., Tsardoulias E., Dimitriou A.G., Symeonidis A.L., Petrou L. (2019). Quantitative and Qualitative Evaluation of ROS-Enabled Local and Global Planners in 2D Static Environments. J. Intell. Robot. Syst..

[5] Campbell S., O’Mahony N., Carvalho A., Krpalkova L., Riordan D., Walsh J. Path planning techniques for mobile robots a review. Proceedings of the 2020 6th International Conference on Mechatronics and Robotics Engineering (ICMRE).

[6] Kelly A. (2013). Mobile Robotics: Mathematics, Models, and Methods.

[7] Chi W., Ding Z., Wang J., Chen G., Sun L. (2021). A generalized Voronoi diagram-based efficient heuristic path planning method for RRTs in mobile robots. IEEE Trans. Ind. Electron..

[8] Hüppi M., Bartolomei L., Mascaro R., Chli M. T-PRM: Temporal Probabilistic Roadmap for Path Planning in Dynamic Environments. Proceedings of the 2022 IEEE/RSJ International Conference on Intelligent Robots and Systems (IROS).

[9] Luan P.G., Thinh N.T. (2023). Hybrid genetic algorithm based smooth global-path planning for a mobile robot. Mech. Based Des. Struct. Mach..

[10] Yang L., Fu L., Li P., Mao J., Guo N., Du L. (2022). LF-ACO: An effective formation path planning for multi-mobile robot. Math. Biosci. Eng.

[11] Mazaheri H., Goli S., Nourollah A. (2024). A survey of 3D Space Path-Planning Methods and Algorithms. ACM Comput. Surv..

[12] Leng S., Sun H. UAV Path Planning in 3D Complex Environments Using Genetic Algorithms. Proceedings of the 2021 33rd Chinese Control and Decision Conference (CCDC).

[13] Chen X., Zheng J., Hu Q. (2023). A Hybrid Planning Method for 3D Autonomous Exploration in Unknown Environments with a UAV. IEEE Trans. Autom. Sci. Eng..

[14] Hua C., Niu R., Yu B., Zheng X., Bai R., Zhang S. (2022). A Global Path Planning Method for Unmanned Ground Vehicles in Off-Road Environments Based on Mobility Prediction. Machines.

[15] Qi W., Xu X., Qian K., Schuller B.W., Fortino G., Aliverti A. (2024). A review of AIoT-based human activity recognition: From application to technique. IEEE J. Biomed. Health Inform..

[16] Jleilaty S., Ammounah A., Abdulmalek G., Nouveliere L., Su H., Alfayad S. (2024). Distributed real-time control architecture for electrohydraulic humanoid robots. Robot. Intell. Autom..

[17] Elbanhawi M., Simic M., Jazar R.N. (2015). Continuous path smoothing for car-like robots using B-spline curves. J. Intell. Robot. Syst..

[18] Blažič S., Klančar G., Loknar M.B., Škrjanc I. (2023). Warehouse Path Planning Using Low-order Bézier Curves with Minimum-Time Optimization. IFAC-PapersOnLine.

[19] Dang C.V., Ahn H., Lee D.S., Lee S.C. (2022). Improved analytic expansions in hybrid A-star path planning for non-holonomic robots. Appl. Sci..

[20] Kim M., Ahn J., Park J. (2022). Targettree-rrt*: Continuous-curvature path planning algorithm for autonomous parking in complex environments. IEEE Trans. Autom. Sci. Eng..

[21] Wang T., Li A., Guo D., Du G., He W. (2024). Global Dynamic Path Planning of AGV Based on Fusion of Improved A* Algorithm and Dynamic Window Method. Sensors.

[22] Adiuku N., Avdelidis N.P., Tang G., Plastropoulos A. (2024). Improved Hybrid Model for Obstacle Detection and Avoidance in Robot Operating System Framework (Rapidly Exploring Random Tree and Dynamic Windows Approach). Sensors.

[23] Wang J., Xu Z., Zheng X., Liu Z. (2022). A fuzzy logic path planning algorithm based on geometric landmarks and kinetic constraints. Inf. Technol. Control.

[24] Carvalho A.E., Ferreira J.F., Portugal D. (2024). 3D traversability analysis and path planning based on mechanical effort for UGVs in forest environments. Robot. Auton. Syst..

[25] Hu J., Hu Y., Lu C., Gong J., Chen H. (2021). Integrated path planning for unmanned differential steering vehicles in off-road environment with 3D terrains and obstacles. IEEE Trans. Intell. Transp. Syst..

[26] Toscano-Moreno M., Mandow A., Martínez M.A., García-Cerezo A. (2023). DEM-AIA: Asymmetric inclination-aware trajectory planner for off-road vehicles with digital elevation models. Eng. Appl. Artif. Intell..

[27] Han L., He L., Sun X., Li Z., Zhang Y. (2023). An enhanced adaptive 3D path planning algorithm for mobile robots with obstacle buffering and improved Theta* using minimum snap trajectory smoothing. J. King Saud-Univ.-Comput. Inf. Sci..

[28] Khaledyan D., Amirany A., Jafari K., Moaiyeri M.H., Khuzani A.Z., Mashhadi N. Low-Cost Implementation of Bilinear and Bicubic Image Interpolation for Real-Time Image Super-Resolution. Proceedings of the 2020 IEEE Global Humanitarian Technology Conference (GHTC).

[29] Liu J., Liu G., Han M., Wan Z., Li Y., Jia X. (2025). Path planning algorithm for articulated loader based on bidirectional Dubins curve. Meas. Sci. Technol..

